# A Comparative Study of Smart THD-Based Fault Protection Techniques for Distribution Networks

**DOI:** 10.3390/s23104874

**Published:** 2023-05-18

**Authors:** Wael Al Hanaineh, Jose Matas, Josep M. Guerrero

**Affiliations:** 1 Electric Engineering Department, Polytechnic University of Catalonia (EEBE-UPC), 08019 Barcelona, Spain; jose.matas@upc.edu; 2 Department of Energy Technology, Aalborg University, 9200 Aalborg, Denmark; joz@et.aau.dk

**Keywords:** fault protection, total harmonic distortion, distribution system, SOGI-FLL

## Abstract

The integration of Distributed Generators (DGs) into distribution systems (DSs) leads to more reliable and efficient power delivery for customers. However, the possibility of bi-directional power flow creates new technical problems for protection schemes. This poses a threat to conventional strategies because the relay settings have to be adjusted depending on the network topology and operational mode. As a solution, it is important to develop novel fault protection techniques to ensure reliable protection and avoid unnecessary tripping. In this regard, Total Harmonic Distortion (THD) can be used as a key parameter for evaluating the grid’s waveform quality during fault events. This paper presents a comparison between two DS protection strategies that employ THD levels, estimated amplitude voltages, and zero-sequence components as instantaneous indicators during the faults that function as a kind of fault sensor to detect, identify, and isolate faults. The first method uses a Multiple Second Order Generalized Integrator (MSOGI) to obtain the estimated variables, whereas the second method uses a single SOGI for the same purpose (SOGI-THD). Both methods rely on communication lines between protective devices (PDs) to facilitate coordinated protection. The effectiveness of these methods is assessed by using simulations in MATLAB/Simulink considering various factors such as different types of faults and DG penetrations, different fault resistances and fault locations in the proposed network. Moreover, the performance of these methods is compared with conventional overcurrent and differential protections. The results show that the SOGI-THD method is highly effective in detecting and isolating faults with a time interval of 6–8.5 ms using only three SOGIs while requiring only 447 processor cycles for execution. In comparison to other protection methods, the SOGI-THD method exhibits a faster response time and a lower computational burden. Furthermore, the SOGI-THD method is robust to harmonic distortion, as it considers pre-existing harmonic content before the fault and avoids interference with the fault detection process.

## 1. Introduction

In recent years, the integration of Distributed Energy Resources (DERs) into Distribution Systems (DSs) has gained considerable attention due to the significant benefits it offers to the overall energy system. DERs, including solar and wind power systems, can contribute to improving energy efficiency and reliability in the distribution grid. They can reduce the dependence on traditional fossil fuel sources, mitigate environmental impacts, and suppsort the integration of new energy technologies, leading to a more sustainable and resilient energy industry [1,2].

However, integrating DERs into DSs requires careful management due to the significant impact it has on the power flow direction. In conventional DSs, the power flow is one-way, but with DER integration, it can be bi-directional, presenting a challenge for the network operator, particularly regarding fault protection [3,4]. Fault protection is a complex issue in DSs, especially with Distributed Generators (DGs), as power flow changes depending on the operational mode. As a result, short-circuit fault current values can fluctuate, resulting in a mismatch between the protective devices (PDs) settings and the actual fault current. This mismatch may lead to miscoordination or even failure of PDs, which can cause equipment damage or power supply loss [5,6].

Conventional protection methods can have limitations when dealing with certain circumstances, such as the inability to identify faults with varying fault resistance, vulnerability to pre-fault load conditions, and susceptibility to noise [7]. Furthermore, these methods can require complex calculations, which could potentially lead to inaccuracies in the fault location prediction [8]. This emphasizes the need for innovative protection strategies that can rapidly and accurately detect and locate faults, thereby reducing power restoration time for customers and improving network performance [9,10,11].

Over the past few years, significant efforts have been made to develop various approaches for detecting and locating faults in DSs with DGs. These approaches can be categorized as conventional protection technologies [12,13,14,15,16,17,18,19], adaptive protection [20,21,22], and harmonics-based protection methods [23,24]. Each method has its own strengths and limitations.

Conventional Overcurrent Relays (OCRs) and differential relays (DRs) are widely used for DS protection. OCRs are mainly employed to detect and prevent faults caused by overloads or short circuits. To ensure sensitivity and selectivity for various system faults, these relays need to be coordinated with other PDs. However, if the network’s topology changes, the settings for OCRs must be updated accordingly [12].

Moreover, when DGs are integrated with the DSs, the fault currents can flow in both directions, which can cause unnecessary disconnections of the circuit breakers (CBs). To solve this issue, directional relays are employed with OCRs, as they together have good current sensitivity and can detect bidirectional power flows effectively [13]. However, using them in all scenarios can be challenging, and the cost of implementation can be high due to the need for additional current transformers (CTs) and CBs [14,15]. In [16], a communication-based directional overcurrent relay was proposed that uses Neural Network (NN) for fast and reliable fault detection. Nevertheless, this method involves complex computations, long training, and difficult implementation, and its reliability is dependent on the quality of the NN training.

In [17,18,19], DRs are capable of detecting faults rapidly and with high sensitivity, regardless of the direction and magnitude of the current flow. However, implementing them can be challenging, expensive, and reliant on communication channels for comparing current quantities. Additionally, they require additional backup protection.

In [20,21], adaptive protection was proposed to automatically adjust the protection’s relay settings to match the power system conditions and determine the operation speed. However, this approach can be costly to implement as it requires advanced digital relays and large computational memory. In [22], another adaptive protection strategy was proposed using both overcurrent and voltage-based methods to set current and voltage thresholds for assessing if a fault occurred within the circuit breaker protection zone. However, this strategy may be undesirable for systems with inverter-interfaced DG where fault current flow is minimal.

In [23,24], harmonic-based methods were proposed, mainly using the harmonic content of the voltage and current in the electrical grid to detect faults. In [23], a new harmonic-based relay was proposed as a cost-effective solution for microgrid (MG) protection. The relay could detect and isolate faults by injecting two different harmonic signals into the grid during a fault, acting as a directional relay without the need for a voltage transformer. However, this method has only been validated for three-phase faults. In [24], the harmonic method was defined using a Fast Fourier Transform (FFT) to obtain the necessary harmonics and THD for each phase of the grid voltage. However, implementing the FFT on a digital signal processor (DSP) supposes a high computational burden, especially when applied to each voltage line of the grid. Furthermore, this method is unable to distinguish between phase-to-phase and phase-to-phase-to-ground faults and exhibits slower tripping responses compared to other methods.

Moreover, there are other methods employed for protection using different techniques such as wavelets [25,26,27], fuzzy logic [28], recursive least square [29,30], differential phase angle [31], s-transform [32], power spectral density and transform [33], deep belief network [34], Hilbert-Huang transform [35]. However, these techniques require a complex computational process and a relatively high cost of implementation.

**Figure 1:**
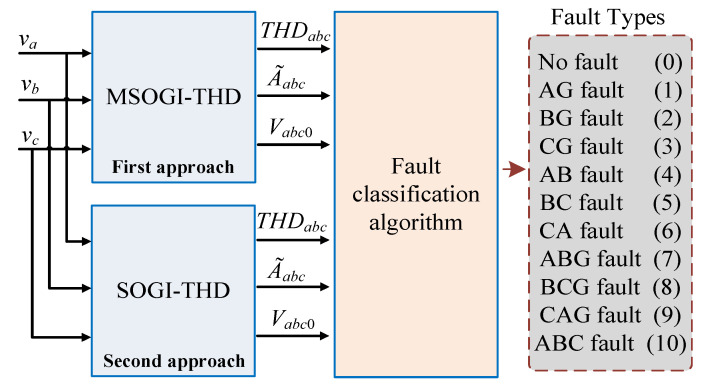
Conceptual block diagram of the two THD protection methods.

To overcome the previous issues, two THD-based protection techniques for detecting and isolating different types of faults (symmetrical and unsymmetrical) in MV DSs were presented in [36,37]. The methods are based on the measurement of the THD levels of the grid voltages, defined as $THD_{abc}$, the estimated amplitude voltages ${\tilde{A}}_{abc}$, and the zero-sequence components $V_{abc0}$. Figure 1 depicts the conceptual diagram of the methods. The first approach in [36] calculates these variables using an MSOGI approach, considering only the fundamental and the *triple-n* harmonics of the grid voltages: the third, sixth, and ninth harmonics. The second approach in [37] uses a SOGI with a few additional math operations to obtain the variables, which was implemented into a DSP from Texas Instruments with a low computational burden.

The purpose of this paper is to assess the effectiveness of these techniques together with traditional overcurrent and differential protections across a range of scenarios. Specifically, the objectives of this study can be summarized as follows:

− The MSOGI-THD and SOGI-THD protection methods are explained and modeled using Matlab/Simulink to automatically detect symmetrical and unsymmetrical fault events in DSs;
− A robust fault detection algorithm for the SOGI-THD is proposed. This approach increases the system’s reliability and accuracy and speeds up the protection system’s response in the event of a fault, regardless of the harmonics present in the grid before the fault. This approach is also computationally efficient;
− A finite state machine has been used to locate and isolate faults in different locations, detect permanent faults, and ignore temporary faults;
− A comparison study between the two THD-based approaches and the conventional methods is proposed under various conditions, such as changing the fault types, DG penetration, fault resistances, and fault locations in the network. Moreover, the study assesses the robustness of these methods against communication delays and the presence of harmonics before fault events. Additionally, the paper evaluates the computational burden of the different THD methods when implemented on a digital processor.

The rest of the paper is structured as follows: Section 2 presents the MSOGI-THD and SOGI-THD approaches. Section 3 presents the proposed fault classification algorithm and the FSM in detail. Simulations and comparisons with OCR and DR systems are carried out to validate the performance of each method in Section 4. Finally, the conclusion is provided in Section 5.

## 2. THD Measurement Methods

The THD is a crucial indicator for evaluating the quality and performance of the power grid. This study utilizes THD as an indicator of system faults and develops fast and sensitive fault protection methods that are both cost-effective and minimize the need for complex equipment. By relying on THD to detect faults, the efficiency and effectiveness of the protection system could be improved while minimizing the risk of costly and time-consuming equipment failures. Overall, this study offers valuable insights into the potential of THD as a fault detection mechanism.

To calculate THD, the square root of the sum of the harmonic components of a signal, squared and divided by the fundamental component, is used. This is per the standard definition outlined in references [38,39] and given by:

(1) $$\mathrm{THD}=\frac{\sqrt{\sum_{h}{\left|A_{h}\right|}^{2}}}{A_{1}}\cdot 100,$$

where *h* and $A_{h}$ are the harmonic order and the amplitude of the *h*-th-harmonic component, respectively, for h≠1, and $A_{1}$ is the amplitude of the fundamental component. In this study, the MSOGI-THD and SOGI-THD methods are employed to acquire the harmonics required for determining the THD.

### 2.1. MSOGI-THD

**Figure 2:**
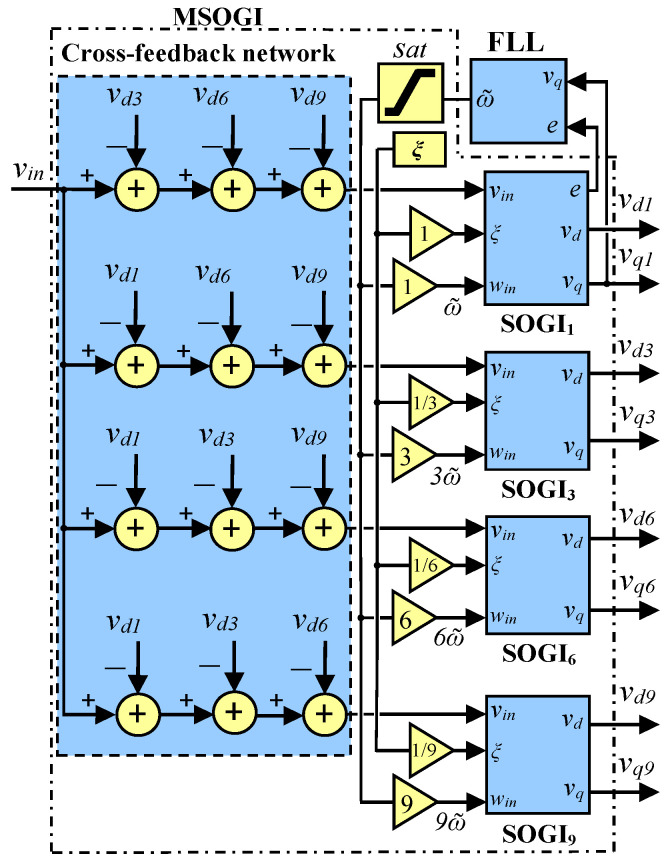
Block diagram of the MSOGI-FLL.

The method described in [36] is used to obtain the harmonic components of the grid in order to calculate the THD. In [36], an MSOGI is employed together with a Frequency Locked-Loop (MSOGI-FLL) to obtain the fundamental and the 3rd, 6th, and 9th harmonics of the grid voltages. The MSOGI uses multiple SOGIs operating in parallel and a cross-feedback cancellation network to remove the unnecessary components from the input signal, ensuring that each SOGI receives only the necessary component. Figure 2 illustrates this configuration, where the input voltage signal is $v_{in}$, the error is e, and the estimated angular frequency is $\tilde{\omega }$.

The MSOGI system uses an FLL to track the operative frequency of the grid. The FLL is linked to the first SOGI, which provides the fundamental component. The FLL delivers $\tilde{\omega }$ to the paralleled SOGIs. The MSOGI can be affected by faults, which distort the estimated$\;\tilde{\omega }$ and cause further distortions to the system. Then, a saturation block is applied to the FLL output to limit the distortion in $\tilde{\omega }$ to be restricted within ±1 Hz of the grid’s nominal frequency. In the MSOGI-FLL, the parameter ξ is set to $1/\sqrt{2}$ to achieve an optimal relationship between rejection to harmonic distortion and transient response speed [40].

In [36], the THD is calculated using the *triple-n* harmonics (i.e., the 3rd, 6th, and 9th) since these harmonics are typically present only in the power inverter’s AC-side neutral point [41]. This ensures that the THD is not influenced by other harmonics that might exist in the grid prior to the fault. When a fault occurs in the grid, there is a sudden drop in the voltage phases, exciting almost all harmonic components. Consequently, the *triple-n* harmonics are also excited, leading to a sharp pulse in the THD. This allows fast detection despite the presence of other harmonics in the grid before the fault. Therefore, during normal operation without faults, the presence of harmonics in the grid does not affect the THD calculation because they are not considered in the computation.

**Figure 3:**
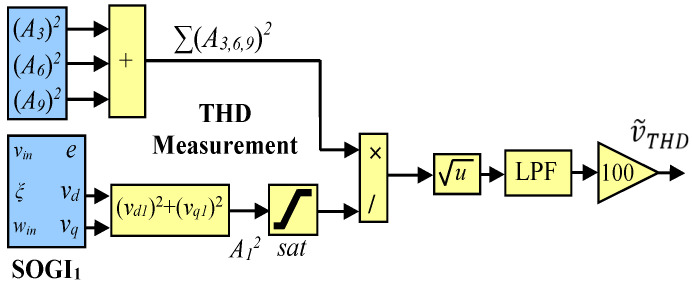
Block diagram of the MSOGI-THD measurement method.

The MSOGI-FLL is used for each phase of the grid voltage and used to determine the fundamental and 3rd, 6th, and 9th amplitudes of the harmonic components by using the in-phase and quadrature-phase outputs of each SOGI as follows:

(2) $${\tilde{A}}_{h}\approx A_{h}=\sqrt{v_{dh}^{2}+v_{qh}^{2}},$$

where h is the index of the harmonic component. To calculate the THD for each phase of the grid, the formula in (1) is used, which involves basic mathematical operations such as summing the squared harmonic components, taking the square root, and dividing, as shown in Figure 3. The resulting THD is denoted as ${\tilde{v}}_{THD}$. It should be noted that multiplication by 100 is only necessary to convert the value to a percentage scale, while saturation is applied to prevent a potential division by 0 during the initial stages of the system. Additionally, a 2nd-order low-pass filter is designed to achieve a balance between the transient response speed and the levels of distortion in the THD signal.

### 2.2. SOGI-THD

The method for measuring THD outlined in [37] is employed in this paper, in which the SOGI approach is used to obtain the harmonic components required for calculating THD [42,43]. The technique described in [37] uses the definition of THD outlined in [38,39], which is calculated using Equation (1). A SOGI-FLL is employed for 1 of the 3 phases of the grid voltage, while only a SOGI is utilized for the remaining two phases, as $\tilde{\omega }$ is provided to them by the FLL stage of the 1st SOGI.

Consider a grid voltage composed of a fundamental and harmonic component as follows:

(3) $$v_{in}\left(t\right)=A_{1}\sin\left(\omega_{i}t\right)+\sum_{h}A_{h}\sin\left(h\omega_{i}t+\varphi_{h}\right),$$

where $\omega_{i}$ and $\varphi_{h}$ represents the grid frequency and the phase angle of the *h*-th-harmonic component, respectively. A SOGI filter is used for each phase to extract its fundamental component,$\;A_{1}$, by using its in-phase and quadrature-phase outputs and (2), while the rest of the harmonic components are given through its error signal, $e\left(t\right)$. Thus, by squaring $e\left(t\right)$ and applying trigonometric identities, it is found that the dc component is equivalent to half the sum of the square of the amplitude of the harmonic components. The dc component can be extracted by applying a low-pass filter with an appropriate cut-off frequency, resulting in the average value of $e\left(t\right)$ as follows:

(4) $$LPF\left(e^{2}\right)=\sum_{h}\frac{A_{h}^{2}}{2},$$

**Figure 4:**
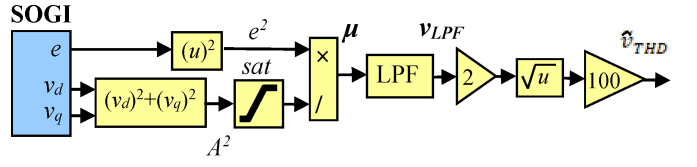
Block diagram of the SOGI-THD measurement method.

Now, the THD is obtained, by multiplying (4) by 2 to remove the 1/2 gain and by taking the square root, as:

(5) $$THD=\sqrt{\frac{2}{A^{2}}LPF\left(e^{2}\right)}\cdot 100=\sqrt{2\cdot LPF\left(\mu \right)}\cdot 100,$$

where $\mu =e^{2}/A^{2}$. Figure 4 depicts the THD’s block diagram. The THD output is denoted as ${\tilde{v}}_{THD}$. Note that multiplication by 100 is used to show the value on a percentage scale, and saturation is employed to prevent an eventual division by 0. The low-pass filter is designed to achieve a balance between speed transient response and distortion levels in the THD signal.

The zero-sequence component is calculated as follows:

(6) $$V_{abc0}=\frac{1}{3}\left(v_{a0}+v_{b0}+v_{c0}\right),$$

**Figure 5:**
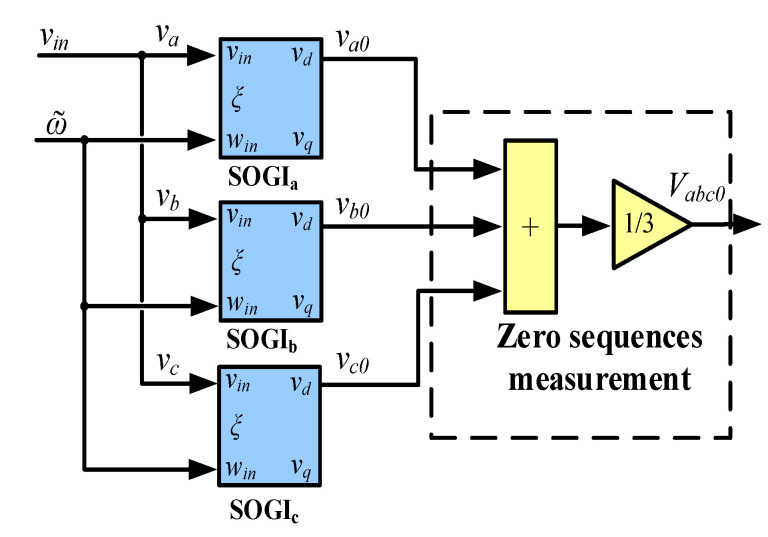
Block diagram for the zero-sequence component measurement, $V_{abc0}$.

And, Figure 5 illustrates the block diagram used to obtain the 0-sequence component. Note that in the SOGI-THD method, only the in-phase voltages of the SOGI used in each phase of the voltage grid are necessary, i.e., $v_{a0}$, $v_{b0}$, and $v_{c0}$. For the case of the MSOGI-THD, only the 1st SOGI of the scheme in Figure 2 is used to achieve the 0-sequence component.

The measurement of THD using the MSOGI-THD and SOGI-THD methods will be able to detect faults since they suppose a sudden sharp change in grid voltages that excites all harmonic components. Therefore, the THD obtained using these methods can be used for a fast of faults, which is the core of the proposed detection algorithms.

As the behavior of $THD_{abc}$, ${\tilde{A}}_{abc}$, and $V_{abc0}$ will differ depending on the type of fault, it is essential to have a fault classification algorithm to accurately identify the type of fault.

## 3. Fault Classification Algorithm

An algorithm has been developed for identifying and detecting faults at each PD location. This algorithm uses measurements of the $THD_{abc}$, ${\tilde{A}}_{abc}$, and $V_{abc0}$ of the three-phase voltage signals to determine the type of fault. For identifying phase-to-phase faults, $V_{abc0}$ will be used.

### 3.1. Proposed System

**Figure 6:**
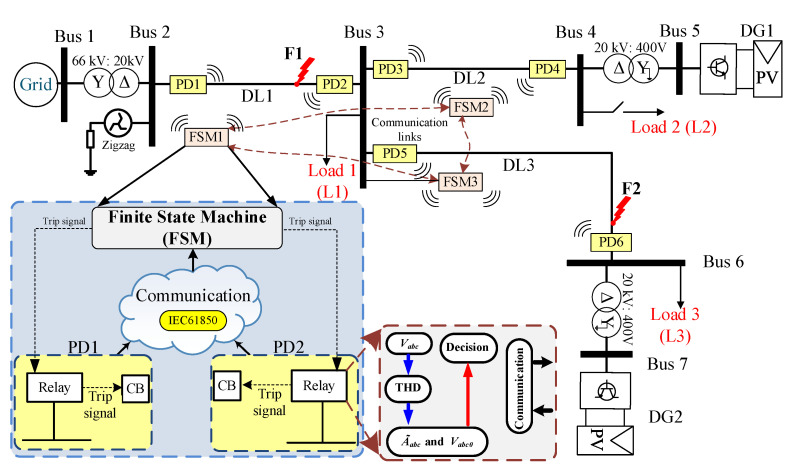
Single line diagram of the proposed network.

Figure 6 illustrates a single-line diagram of the DS used for testing the methods, and the system parameters are provided in Table 1. The diagram consists of a main grid and multiple DGs connected to various buses. The high-voltage (HV) grid has a rated voltage of 66 kV and a rated power of 25 MVA, and it is connected to three Distribution Lines (DLs; DL1, 2, and 3) through a step-down HV/MV transformer. This transformer is configured in a star/delta (YNd11) setup, which means its ground connection can significantly impact the short-circuit current in the network and, therefore, the behavior of the protection system. To overcome this issue, a zig-zag transformer is used to create an isolated neutral in the power system. This common configuration provides a balanced and stable system even in the presence of ground faults. In this work, the zig-zag transformer is connected to the delta side of the voltage transformer, which is grounded through zig-zag at Bus 2, as shown in Figure 6 [44]. Each DL has two PDs located on either side of the line, with an FSM intended for fault isolation. The PDs contain a fault detection relay and circuit breaker that trip and isolate the line based on a message from the FSM when a fault occurs. Different local loads (L1 to L3) are connected to the end of each DL. Two DGs (DG1 and 2) are connected, through an MV/LV transformer, to different locations of the system. Loads and DGs are connected to the low-voltage side using a delta/star grounded (Dyn11) configuration [44]. Communication channels are employed between the PDs and the FSM, and between the FSMs themselves, for transmitting trip signals to coordinate and isolate faulted locations. The proposed approach’s behavior will be tested under various scenarios in different locations, defined as F1 and F2, in Figure 6.

### 3.2. Fault Classification Algorithm Stages

In this work, the algorithm uses three stages to perform a rapid and secure decision to detect and identify types of faults that may occur in the grid.

#### 3.2.1. Pre-Processing Stage

This stage involves measuring the three-phase voltages,$\;v_{abc}$, at each PD in the time domain. From these measurements, the $THD_{abc}$, ${\tilde{A}}_{abc}$, and $V_{abc0}$ are calculated using the MSOGI-THD and SOGI-THD approaches, as previously explained.

#### 3.2.2. Fault Detection Stage

1. MSOGI-THD

In this case, the fault is detected using a threshold, $\alpha_{MSOGI}$. The transient response induced by the fault in $THD_{abc}$ is compared with $\alpha_{MSOGI}$, and the detection is activated when $THD_{abc}>\alpha_{MSOGI}$. $\alpha_{MSOGI}$ is set to 5%, which is the recommended level for voltage harmonic distortion for DS in the IEEE standard 519-2014 [45].

2. SOGI-THD

**Figure 7:**
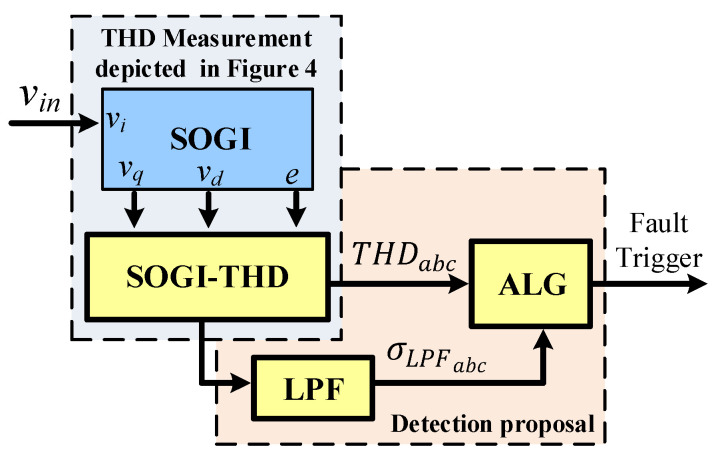
Adaptive SOGI-THD fault detection scheme using $THD_{abc}$ and $\sigma_{LPF_{abc}}$.

In this case, the fault is detected using the $THD_{abc}$ transient response and low-pass filtering of THD, noted as $\sigma_{LPF_{abc}}$, which allows the detection of faults even when there is harmonic distortion in the grid. The design of this proposal is illustrated in Figure 7.

The detection is done based on the difference between $THD_{abc}$ and $\sigma_{LPF_{abc}}$. at the precise instant a fault occurs. This difference is known as $\alpha_{abc}$:

(7) $$\alpha_{abc}=THD_{abc}-\sigma_{LPF_{abc}},$$

where $\alpha_{abc}$ represents the difference calculated for each phase. The presence of a fault is detected when $\alpha_{abc}$ exceeds a predefined threshold,$\;\alpha_{abc}\geq \alpha_{o}$. In this case,$\;\alpha_{o}$ was set to 25% to ensure a sufficient margin for avoiding false triggering caused by unexpected transients. The $\sigma_{LPF_{abc}}$ was obtained by applying a low-pass filter with an appropriate cutoff frequency to $THD_{abc}$, thus obtaining its average.

**Figure 8:**
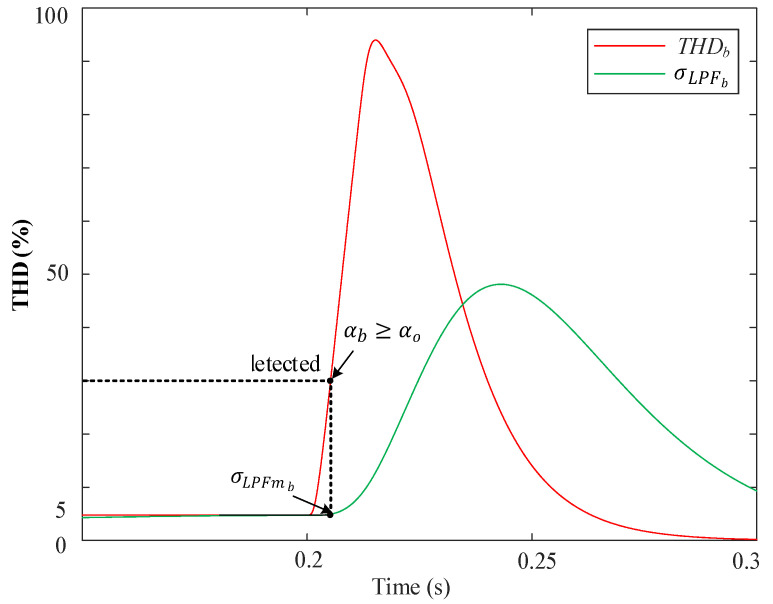
An example of the behavior of $THD_{b}$ and $\sigma_{LPF_{b}}$ during a fault of phase “b” with the proposed fault detection algorithm.

Figure 8 depicts the behavior of the THD for phase “*b*” during a fault at 0.2 s, and simultaneously, there is a fifth harmonic with a 5% amplitude distortion in the grid voltage. Prior to the fault, the harmonic had been there for a sufficient duration to make the output of the LPF equal to the input, so $\alpha_{b}=0$. When a fault occurs, a sudden, sharp peak-pulse waveform is produced in $THD_{b}$ that disappears over time, following an exponential decay pattern. This transient is filtered by the low-pass filter ($\sigma_{LPF_{b}}$) which has a much slower response than the $THD_{b}$ and takes a lot of time to be observed at its output. However, at the moment of the fault, $\sigma_{LPF_{b}}$. remains close to the $THD_{b}$ level that existed prior to the fault. Thus, the difference, $\alpha_{b}$, closely corresponds to the difference between the actual $THD_{b}$ and the past $THD_{b}$ level that the grid had at the moment of the fault. Therefore, the utilization of the low-pass filter in this manner allows for the detection of sudden changes in $THD_{b}$ caused by faults, regardless of the prior harmonic distortion levels that might be present in the grid voltage. Upon detection, the algorithm reads and records the value of $\sigma_{LPF_{b}}$, referred to as $\sigma_{LPFm_{b}}$, which will be used later for the identification process.

#### 3.2.3. Fault Identification Stage

In this stage, the identification process, in case of using any of the two THD approaches, is done based on the behavior of ${\tilde{A}}_{abc}$ and $V_{abc0}$, depending on the fault case.

The MSOGI-THD approach uses a threshold named Δv for comparing with ${\tilde{A}}_{abc}$. Δv is set to 7.5%, which is the acceptable range for grid voltage drop at the medium voltage level as recommended by the technical requirements in the Spanish grid code for reliable energy integration [46]. In contrast, the SOGI-THD approach employs two thresholds for identifying faults, $\sigma_{LPFm_{abc}}$ for comparing with $THD_{abc}$, which should be verified as $THD_{abc}>\sigma_{LPFm_{abc}}$ upon detection, and Δv, for comparing with ${\tilde{A}}_{abc}$. In this case, Δv is also set to 7.5% [46].

**Figure 9:**
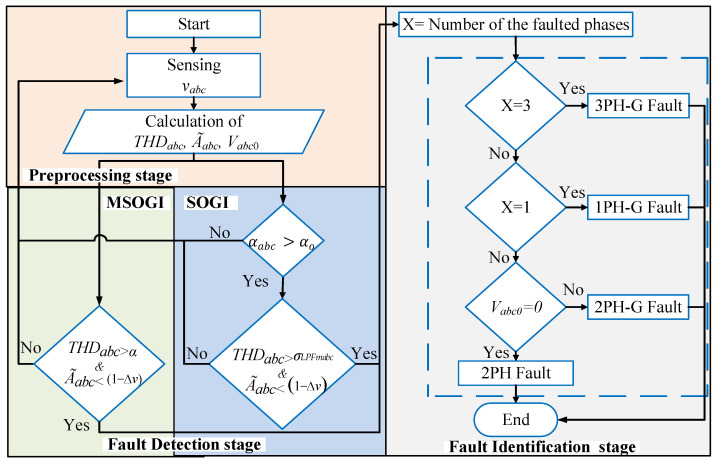
Flowchart of the fault classification algorithm.

In both THD approaches, during a fault event, a sudden drop in ${\tilde{A}}_{abc}$ is produced due to the fall in the voltages of the faulted phases, which makes any of the estimated phases be below the threshold, i.e., ${\tilde{A}}_{abc}<\left(1-\Delta v\right)$ pu. Then, this condition is considered a fault event. At this point,$\;V_{abc0}$ is used by both methods (THD approaches) to differentiate between a phase-to-phase (2PH) and phase-to-phase-ground (2PH-G) fault, as the initial conditions for both types of faults are the same. In the event of a 2PH fault, the zero-sequence voltages are zero, while they are non-zero in the case of a 2PH-G fault [47]. Therefore, $V_{abc0}$ is utilized to determine the type of fault and adopt a decision. The faults were grouped into 11 categories, numbered from zero to 10, see Figure 1. Figure 9 depicts the fault classification algorithm structure.

## 4. Finite State Machine

In the grid-line diagram of Figure 6, three FSMs have been defined to locate and isolate faults in different locations of the DS. Fault isolation refers to the process of disconnecting a faulted part of a system, in this case, DL, while keeping the rest of the system operational by maintaining power to the rest of the DLs and the corresponding loads they serve [47]. This allows for repairs to be made to the faulted section without disrupting power to the entire system or loads. Fault isolation is an important aspect of power system protection and helps to minimize the impact of faults on the system and the customers.

In addition, the FSMs are designed to detect permanent faults and ignore temporary faults, thus avoiding unnecessary tripping of protection devices, which can save time and resources. Note that a permanent fault is expected to persist, while a temporary fault is expected to clear on its own without any protection action. The duration of a temporary fault is usually less than 100 ms [48,49].

**Figure 10:**
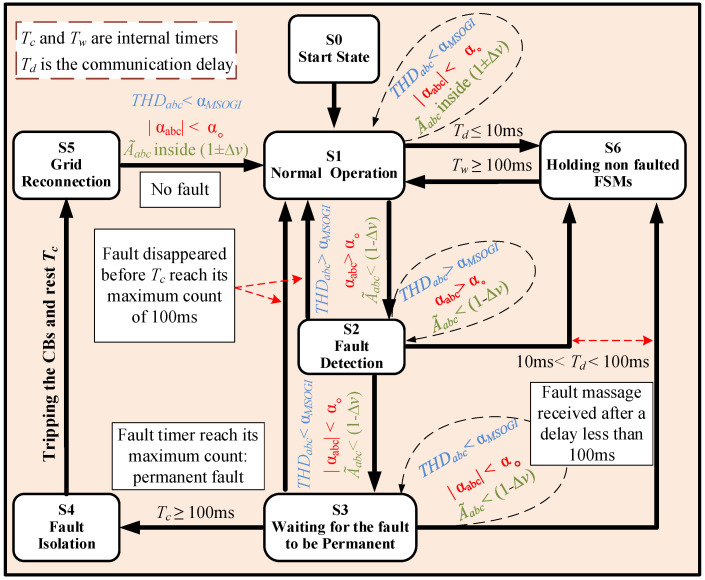
Scheme of the proposed FSM.

In this system, each FSM is allocated to a DL with its two PDs and comprises six distinct states, as depicted in Figure 10. The figure provides a description of the FSM for the two THD approaches, where the blue color denotes the MSOGI-THD, the red color represents the SOGI-THD, and the green color signifies the common conditions of both approaches.

### 4.1. State S1: Normal Operation

The FSM typically begins in state S1 and stays there, while the system operates within a boundary of 1 ± ∆v pu around nominal values and $THD_{abc}<\alpha_{MSOGI}$ for the MSOGI-THD and $\left|\alpha_{abc}\right|<\alpha_{o}$ for the SOGI-THD. Therefore, at S1, the FSM is waiting for a fault.

### 4.2. State S2: Fault Detection

When a fault occurs, the PD in charge of a DL detects it by observing $THD_{abc}>\alpha_{MSOGI}$ and ${\tilde{A}}_{abc}<\left(1-\Delta v\right)$ pu for the MSOGI-THD or $\alpha_{abc}\geq \alpha_{o}$ for the SOGI-THD, and ${\tilde{A}}_{abc}<\left(1-\Delta v\right)$ pu. At this point, a Wi-Fi protocol communication [50] is initiated between the PD and the FSM of a faulted DL to send a fault message to the FSM. Upon receiving the message, the FSM transitions to state S2.

It is important to mention that the detection time of a fault by the algorithm inside the PD of a DL is influenced by the distance between the PD and the fault location. Specifically, the PD closest to the fault is expected to detect the fault in the shortest time. On the other hand, the Wi-Fi communication delay for a fault message to be transmitted will be small enough, less than 1 ms [51]. This gives the FSM a chance to have a high level of responsiveness to quickly locate the fault and adopt the decision to start the isolation process.

In the event of a communication failure, the PD located at the other end of the faulted DL is responsible for transmitting the fault message to the FSM. This is because it is the second-fastest PD to detect the fault. As a result, the FSM of the faulted DL that is closest to the fault will generally locate the fault more quickly than the other FSMs. This feature is utilized to achieve faster and more efficient fault isolation, thereby minimizing the impact of the fault.

Now, once the faulted DL’s FSM enters S2, after receiving a fault detection message from the PD in charge, a timer named $T_{c}$ is started inside to measure the fault duration. Additionally, a new fault signal is sent to the other FSMs via communication links, advising them of the event and allowing them to stop their processes. For example, if a fault occurs at DL1, FSM1 will detect the fault faster than the other FSMs.

The variability in the transmission time of a fault message between FSMs can be attributed to the communication technology employed and additional delays, referred as $T_{d}$. This system utilizes the IEC 61850 protocol, which is estimated to have a delay of 10 ms [52]. As a result, this protection system is reliant on a communication delay between the FSMs to coordinate and operate effectively. However, delays between 10 ms and 100 ms can occur, which may cause an FSM to receive a fault message during its process. In cases where the delay exceeds 100 ms, the FSM may make an independent decision, potentially resulting in the tripping of breakers.

Meanwhile, in S2, if the fault disappears, as in a temporary fault, then $THD_{abc}<\alpha_{MSOGI}$ for the MSOGI-THD, or $|\alpha_{abc}|<\alpha_{o}$ for the SOGI-THD, and ${\tilde{A}}_{abc}$ will return to its normal value. The FSM will then return to its normal operation state (S1). Additionally, if the FSM receives a fault message from the other FSMs, it will transition to state S6, which is used to hold non-faulted FSMs.

The THDs exhibit a spike when a fault occurs, which gradually decreases over time and eventually disappears. Therefore, whenever $THD_{abc}<\alpha_{MSOGI}$ for the MSOGI-THD, or $|\alpha_{abc}|<\alpha_{o}$ for the SOGI-THD, while ${\tilde{A}}_{abc}<\left(1-\Delta v\right)$ pu still holds, the FSM enters state S3 to check for the permanence of the fault. This peak behavior that exponential decay can be observed in the figures presented in the results section of the paper.

### 4.3. State S3: Waiting for the Fault to Be Permanent

In this state, the timer $T_{c}$ continues to count up, and once it reaches 100 ms, the FSM transitions to state S4 for fault isolation. However, if the fault disappears, i.e., ${\tilde{A}}_{abc}$ returns to its normal value before $T_{c}$ reaches 100 ms, the FSM returns to S1 (normal operation) without tripping the circuit breakers. In the event that the FSM receives a fault message during state S3, and $T_{c}$ is less than 100 ms, it transitions to state S6, indicating that the fault is not located within the FSM’s protection area.

### 4.4. State S4: Fault Isolation

The FSM enters this state only when $T_{c}$ reaches 100 ms, indicating that the fault is permanent. Therefore, the FSM sends an active trip signal to the nearest circuit breaker to isolate the fault. Furthermore, if any of the non-faulted FSMs also enter state S4, this implies that they did not receive a fault message from the faulted FSM due to a communication loss. After the tripping, $T_{c}$ is reset to 0, and the FSM goes to state S5.

### 4.5. State S5: Grid Reconnection

In this state, the FSM waits for the grid operator or maintenance personnel to reconnect the line and return the FSM to its normal operation state (S1).

### 4.6. State S6: Holding Non-Faulted FSMs

Finally, the FSM transitions to this state upon receiving a fault signal from a faulted FSM in any of the S1, S2, or S3 states. At S6, the FSM keeps waiting until the fault either disappears or is isolated. This waiting action is accomplished using a count-up timer called $T_{w}$. When $T_{w}$ reaches 100 ms, the FSM returns to its normal operating state (S1).

## 5. Results

In this section, a comparison is conducted first between the MSOGI-THD and SOGI-THD protection methods and then with other conventional protection methods. The goal is to evaluate the performance and effectiveness of each method in detecting and isolating faults in the proposed DS (see Figure 6). The methods are analyzed using MATLAB/Simulink software under various scenarios, including changes in DG penetration, fault type, fault resistance, and location, as well as in case of pre-existing harmonics and additional communication delay. The parameters of the network in Figure 6 are shown in Table 1.

### 5.1. DG Penetration Protection Test

In this section, the two protection methods are evaluated in the event of a fault occurring at 0.2 s with varying levels of DG penetration. The conditions used in the evaluation are given in Table 2.

#### 5.1.1. Three-Phase Fault (3PH-G) at F1

**Figure 11:**
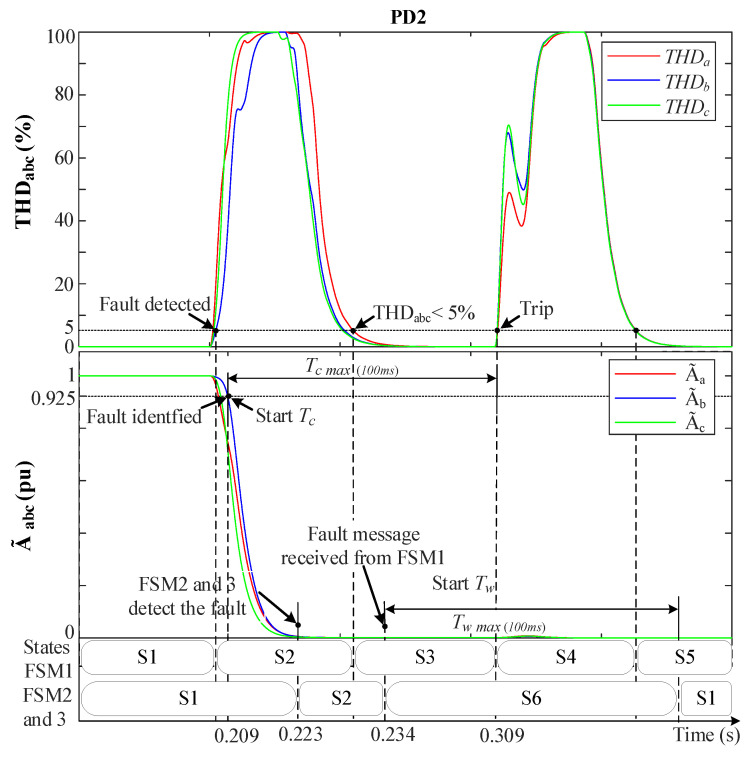
MSOGI-THD protection method behavior during a 3PH-G fault with 2DGs at F1. Upper: $THD_{abc}$. Lower: ${\tilde{A}}_{abc}$.

**Figure 12:**
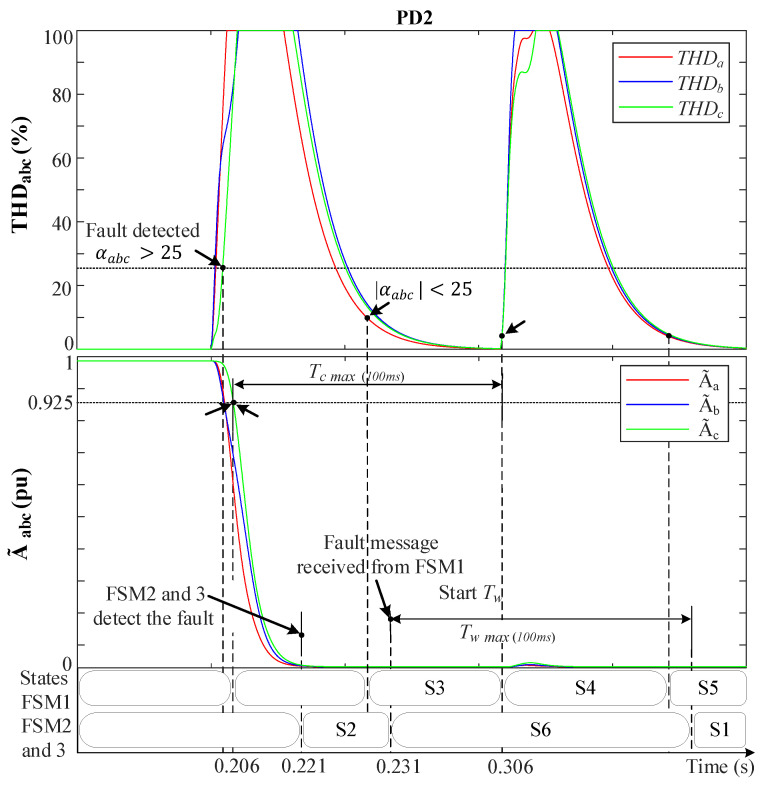
SOGI-THD protection method behavior during a 3PH-G fault with 2DGs at F1. Upper: $THD_{abc}$. Lower: ${\tilde{A}}_{abc}$.

Figure 11 and Figure 12 depict the behavior of the MSOGI-THD and SOGI-THD protection methods, respectively, during a 3PH-G with 2 DGs at F1; see Figure 6. Notice in both figures how $THD_{abc}$ increases abruptly at 0.2 s while ${\tilde{A}}_{abc}$ drops toward 0 pu due to the low fault resistance (r=0.001 Ω). In Figure 11, the fault is detected at 0.2065 s when $THD_{abc}>5\%$. At 0.209 s, the condition ${\tilde{A}}_{abc}<0.925$ is met; therefore, the fault is identified as an (ABC) fault.

In Figure 12, the fault is detected at 0.204 s when $\alpha_{abc}>25$ and $\sigma_{LPF_{abc}}$ is read and stored in memory ($\sigma_{LPFm_{abc}}$). At 0.206 s, the conditions ${\tilde{A}}_{abc}<0.925$ and $THD_{abc}>\sigma_{LPFm_{abc}}$ are met; therefore, the fault is identified as an (ABC) fault. In both methods, once PD2 detects and identifies the fault, a fault message is sent to FSM1 using wireless communication channels. It is worth noting that if PD2 fails for any reason, or in the case of communication problems, PD1 will be the second fastest to be in charge of detecting the fault.

When FSM1 receives the fault message from PD2, it immediately moves to S2 and sends a fault message to other non-faulted FSMs while starting a count-up on its own $T_{c}$ timer. In this fault-case scenario, an extra delay of 25 ms has been intentionally added to the FSM’s communication links to show its effect on the protection system. As a result, the non-faulted FSMs detect the fault and move to S2 before receiving the message. For instance, in Figure 11, FSM2 and 3 detect the fault (i.e., move to S2) after 23 ms due to the distance between the fault location and the PDs of DL2 and 3.

Once the FSMs receive the message, 25 ms later due to the delay, they transition from S2 to S6 (hold state), and $T_{w}$ starts counting up to wait for the fault to be isolated. The $THD_{abc}$’s behavior in FSM1 has a peak that decays exponentially to zero after a short time. Then, when $THD_{abc}$ returns to be below 5% in Figure 11 or when the absolute value of $\alpha_{abc}$ is less than the threshold $\alpha_{o}$ ($\left|\alpha_{abc}\right|<\alpha_{o})$ in Figure 12, while ${\tilde{A}}_{abc}<0.925$ pu still holds, FSM1 transitions from S2 to S3, and $T_{c}$ continues counting up. Upon reaching $T_{c}=100\mathrm{ms}$, the fault is declared permanent, and FSM1 moves from S3 to S4, causing the circuit breakers in DL1 to trip and isolate the fault. Additionally, $T_{c}$ is reset to 0, and FSM1 transitions from S4 to S5 to wait for actions to reconnect DL1 to the grid. Note that in FSM1, a momentary disruption in the grid occurs when transitioning from S3 to S4 due to DL1 disconnection, causing $THD_{abc}$ to spike again. However, this does not affect the FSM since it is in state S4 and does not consider THD. In non-faulted FSMs, once $T_{w}$ reaches 100 ms, they return to S1 (normal operation).

**Figure 13:**
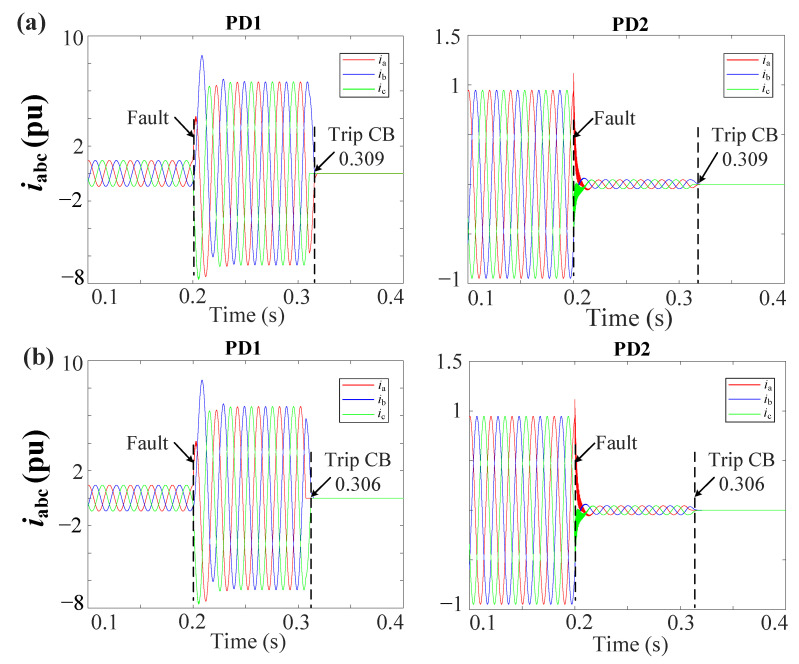
$i_{abc}$ currents for the grid during a 3PH-G fault with 2DGs at DL1 using (**a**) MSOGI-THD and (**b**) SOGI-THD.

Figure 13a,b shows the grid currents ($i_{abc}$) during a 3PH-G fault with 2DGs at DL1 for the MSOGI-THD and SOGI-THD protection systems, respectively. Note in Figure 13a that, due to the tripping signal sent by FSM1, both PD1 and PD2 of DL1 are tripped at 0.309 s. In Figure 13b, note that the PDs trip at 0.306 s.

#### 5.1.2. Phase-to-Phase Fault (2PH) at F2

**Figure 14:**
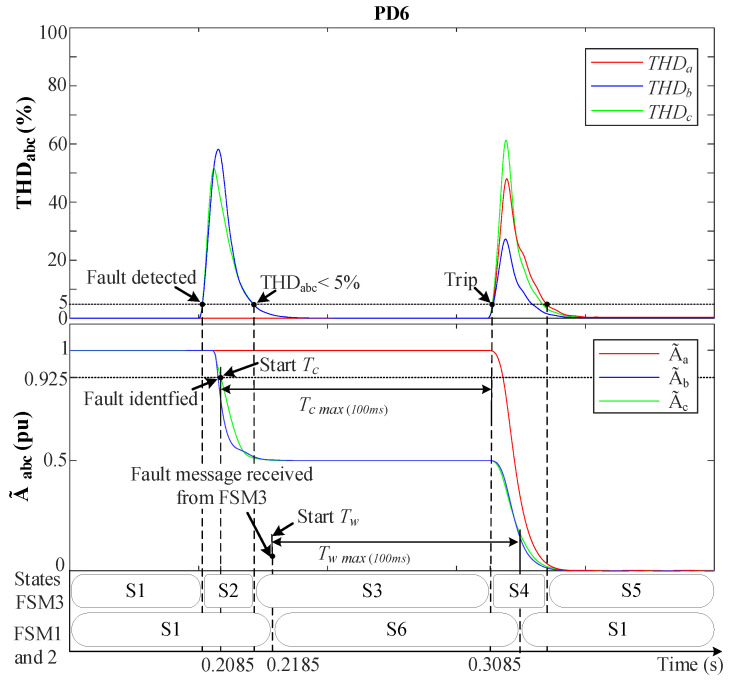
MSOGI-THD protection method behavior during a 2PH fault without DGs at F2. Upper: $THD_{abc}$. Lower: ${\tilde{A}}_{abc}$.

**Figure 15:**
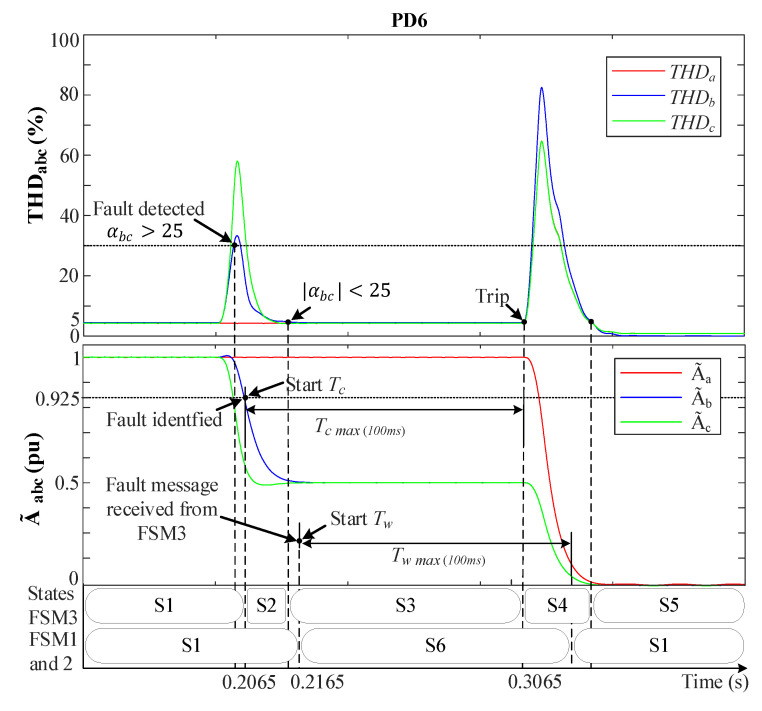
SOGI-THD protection method behavior during a 2PH fault without DGs at F2. Upper: $THD_{abc}$. Lower: ${\tilde{A}}_{abc}$.

Figure 14 and Figure 15 demonstrate the behavior of the MSOGI-THD and SOGI-THD protection methods, respectively, during a 2PH event between phases *b* and *c* in the absence of DGs at F2, as shown in Figure 6. During this test, a 5th harmonic distortion was introduced in the grid before the fault. Figure 14 indicates that $THD_{abc}$ remains unaffected by the 5th harmonic distortion because the MSOGI-THD design does not consider this harmonic in his scheme. In contrast, Figure 15 shows that the SOGI-THD detected the 5th harmonic distortion. However, since $\alpha_{abc}<25$, it will not be considered a fault, and the algorithm will not trigger any protective action.

It is worth noting that, at the time of the fault (0.2 s) in both Figures, only $THD_{bc}$ increases abruptly, whereas $THD_{a}$ remains at 0%. Whereas ${\tilde{A}}_{bc}$ drops towards 0.5 pu due to the absence of the ground connection and fault resistance (r=0.1 Ω), while ${\tilde{A}}_{a}$ remains unaffected (1 pu). In Figure 14, the fault is detected at 0.2055 s when $THD_{bc}>5\%$. At 0.2085 s, the condition ${\tilde{A}}_{bc}<0.925$ is met and $V_{abc0}$ is checked to be zero, leading to the identification of the fault as a (BC) fault.

Figure 15 shows that the fault is detected at 0.204 s when $\alpha_{bc}>25$ and $\sigma_{LPF_{bc}}$ is read and stored in memory ($\sigma_{LPFm_{bc}}$). At 0.2065 s, the algorithm identifies the fault as a (BC) type since the ${\tilde{A}}_{bc}<0.925$, $THD_{bc}>\sigma_{LPFm_{bc}}$, and $V_{abc0}=0$ conditions are met. It is worth mentioning that, in both protection methods, after PD6 detects the fault, a fault message is transmitted wirelessly to FSM3.

Upon receiving the fault message from PD6, FSM3 enters state S2 and sends a notification to the other non-faulted FSMs while initiating the countdown on its $T_{c}$ timer. These FSMs receive the message after 10 ms due to the normal delay of the communication links and move from S1 to S6 (hold state), and $T_{w}$ starts counting up. The $THD_{abc}$’s behavior of FSM3 suffers from a peak that decays exponentially to zero in a short time. Then, when $THD_{bc}$ returns to $THD_{bc}<5$% in Figure 14 or when $\left|\alpha_{bc}\right|<\alpha_{o}$ in Figure 15 while ${\tilde{A}}_{bc}<0.925$ pu still holds, FSM3 goes from S2 to S3, and $T_{c}$ continues counting up. Whenever $T_{c}$ reaches 100 ms, the fault is considered permanent, and FSM3 moves to the fault isolation state (S4), sending a signal to trip the circuit breakers in DL3. Then, $T_{c}\;$is reset, and FSM3 moves to S5, where it waits for reconnection actions. While FSM3 is in state S4, all THD signals experience a spike resulting from DL3 disconnection, which causes an abrupt change in the grid voltages. However, since FSM3 is in S4, this does not affect its operation. At the non-faulted FSMs, the timer $T_{w}$ continues counting up, and once it reaches 100 ms, they return to the normal operation state (S1).

**Figure 16:**
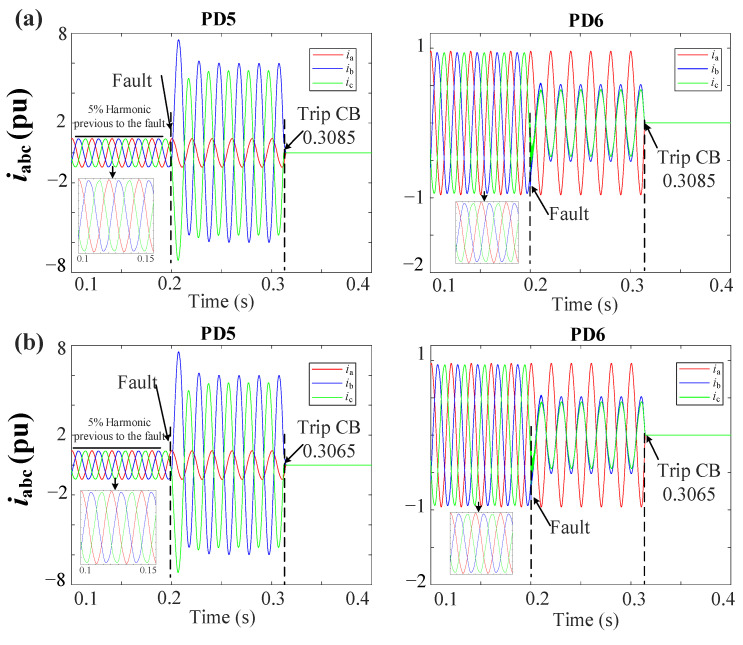
$i_{abc}$ currents for the grid during a 2PH fault without DGs at DL3 using (**a**) MSOGI-THD and (**b**) SOGI-THD.

Figure 16a,b shows the grid currents ($i_{abc}$) during a 2PH fault without DGs at DL3 for the MSOGI-THD and SOGI-THD protection systems, respectively. It can be observed that a fifth harmonic of 5% is present before the fault. Note in Figure 16a that, due to the tripping signal sent by FSM3, both PD5 and PD6 of DL3 are tripped at 0.3085 s. In Figure 16b, note that the PDs trip at 0.3065 s.

### 5.2. Comparison with Different Protection Methods

The system presented in Figure 6 was used to test the effectiveness of the conventional overcurrent and differential protections [53] and compare them with the MSOGI-THD and SOGI-THD methods. Recent research shows that integrating DGs into the power grid poses a threat to conventional protections, particularly in complex systems with multiple protection devices. As a result, coordinating the Overcurrent Relays (OCRs) can be difficult, leading to false tripping and unnecessary service interruptions [54].

The Differential Relay (DR) is a commonly employed protection that depends on the differential current value to address the issue of bidirectional power flow in a DS. Nevertheless, if the current transformers are saturated or not properly configured, the DR may experience tripping problems. Moreover, the settings of both the OCR and DR must be updated due to the grid’s changing circumstances [55,56,57].

#### 5.2.1. Fault Detection Performance Analysis for the Protection Approaches

A 3PH-G (ABC) fault with 2DGs located at DL1 in F1 is considered in this section at 0.2 s with a fault resistance of 0.001Ω to compare the performance of the OCR and DR with the results of the proposed THD methods presented in Section 5.1.1. Table 3 and Table 4 show the settings used for the OCR and DR, respectively. It’s worth noting that the OCR was designed with the IEEE extremely inverse curve, which ensures the fastest disconnection in comparison with the other curves [58].

**Figure 17:**
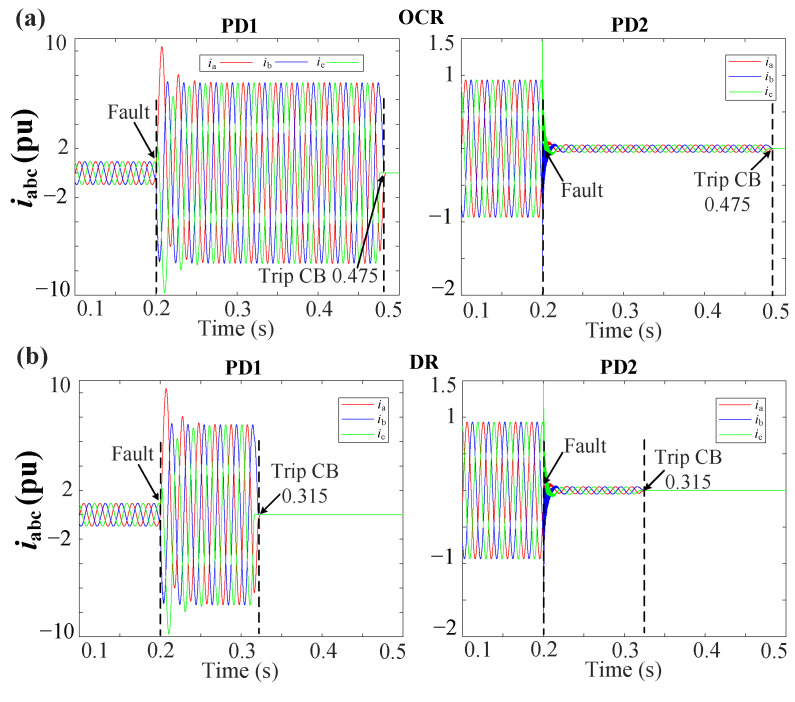
$i_{abc}$ currents for the grid during a 3PH-G fault with 2DGs at DL1 using (**a**) OCR and (**b**) DR.

The performance of the OCR and DR protection methods is shown in Figure 17. The OCR and DR had been designed to take action after a delay of 100 ms of the fault detection for simplicity of comparison.

Table 5 shows the fault detection and clearing times for each method. Note that the DR and OCR of DL1 had cleared the fault at 0.315 s and 0.4750 s, respectively, which, considering the 100 ms, means that the DR takes 15 ms to detect the fault, whereas the OCR takes 175 ms. Figure 17 indicates that utilizing the OCR protection method is not advisable, as it allows the fault to continue for several cycles, posing a risk of equipment damage. It can be inferred that the DR, MSOGI-THD, and SOGI-THD methods can rapidly isolate the faulted DL in the system. Nevertheless, the SOGI-THD protection approach demonstrated the fastest response, clearing the fault at 0.306 s, meaning that it takes only 6 ms for detection, as shown in Figure 13b.

#### 5.2.2. Different Fault Resistance Protection Tests

**Figure 18:**
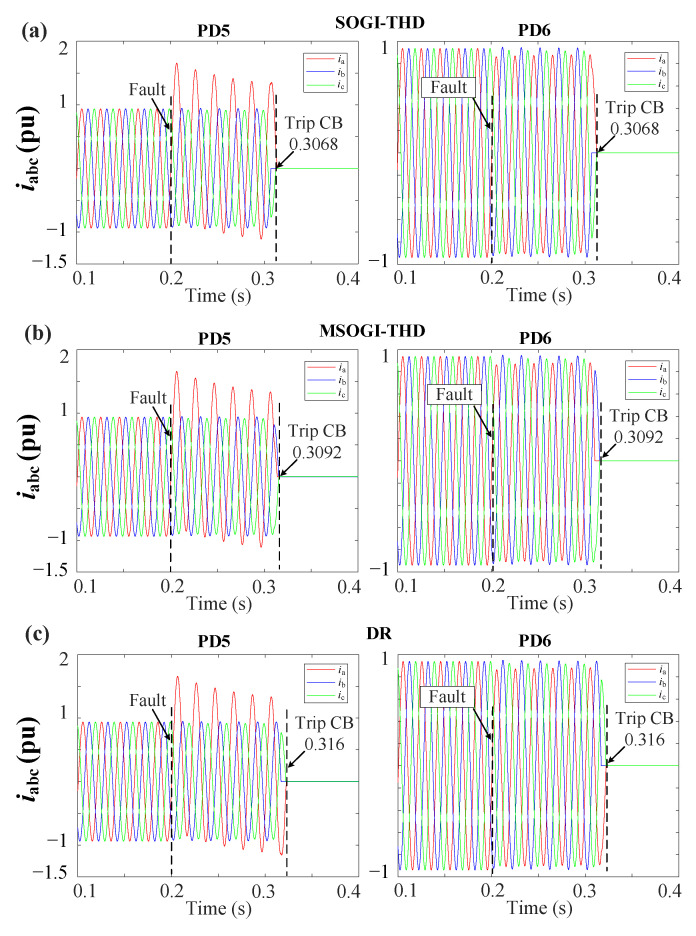
$i_{abc}$ currents for the grid during a 1PH-G fault with 2DGs at DL3 using (**a**) SOGI-THD, (**b**) MSOGI-THD, and (**c**) DR protection methods.

Changing the fault impedance can pose a challenge in detecting faults in distribution networks for many protection techniques. Therefore, this section explores the behavior of the protection methods when an unsymmetrical 1PH-G fault with the 2DGs occurs at the F2 location in DL3 at 0.2 s; see Figure 6. The fault resistance is changed to r=6 Ω. Figure 18 shows the performance of the DR, MSOGI-THD, and SOGI-THD methods during the fault. It can be observed that the SOGI-THD is the fastest at clearing the fault. The SOGI-THD, MSOGI-THD, and DR clear the fault at 0.3068 s, 0.3092 s, and 0.316 s, respectively.

**Figure 19:**
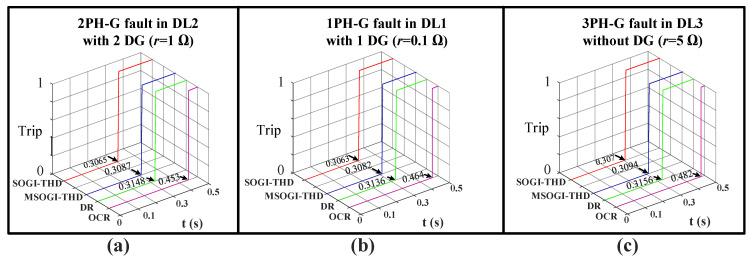
Fault clearing times of the OCR, DR, MSOGI-THD, and SOGI-THD protection methods during different faults types and resistances (**a**) 2PH in DL2, (**b**) 1PH in DL1, (**c**) 3PH-G in DL3.

The protection methods were tested under various fault resistances, types, locations, and DG penetrations. The results consistently showed that in all scenarios, the SOGI-THD method cleared the fault faster than the other ones, as seen in Figure 19.

#### 5.2.3. Different THD Methods Computational Burden Assessment

The THD protection method explained in [24] is compared with the SOGI-THD and MSOGI-THD methods to validate and emphasize the advantages of the proposed approaches. In [24], a Fast Fourier transform (FFT) was used to achieve the required harmonics and THD for each phase of the voltage signal and define the protection algorithm.

According to [36,43], the SOGI-FLL and FFT methods were implemented into a DSP, and the computational burden was computed in the number of processor cycles (c) used to compute the SOGI, the FLL, and the FFT blocks. In [43], it was reported that the SOGI needs 149c, the FLL 49c, and the FFT 7019c per phase. Table 6 shows the number of SOGIs and the processor cycles required for executing each method. It can be seen from Table 6 that the SOGI–THD method requires the lowest number of SOGIs and processor cycles when it is executed in the DSP. This justifies the fact that it is much more computationally affordable without affecting the THD calculation or the operation of the protection system.

Additionally, the THD protection techniques proposed in this study are compared alongside other methods in similar conditions. Table 7 compares the methods based on their response speed, accuracy, cost of implementation, the use of inverter-based systems, communication needs, and the grid configuration used for testing. Table 8 presents a summary of the tripping times of these methods and their respective advantages and drawbacks. It can be concluded that the SOGI-THD approach may be a feasible solution for ensuring reliable and fast protection under various conditions when using communication lines.

## 6. Discussion

This study aims to compare the effectiveness of two THD-based protection methods, MSOGI-THD and SOGI-THD, against traditional overcurrent and directional protection methods in a radial distribution system (DS) that includes distributed generation (DG) connections. The analysis takes into account various fault scenarios, types of faults, fault resistances, DG penetrations, and fault locations. The impact of communication delays and the presence of distorting harmonics on the effectiveness of these protection methods have also been examined.

The results indicate that both MSOGI-THD and SOGI-THD protection methods are highly effective in detecting faults in distribution systems with varying levels of DG penetration and fault resistance. In addition, these methods are capable of differentiating between actual faults and harmonic distortions that may exist in the system prior to a fault, which helps prevent unnecessary protective actions. Moreover, the results showed that communication delays had little impact on the performance of the protection process, which points out that the THD-based protection methods may be well suited for use in large-scale power grids where communication delays are common.

In addition, the comparison found that the THD-based protection methods performed better than traditional OCR and DR protection methods in terms of response time and efficiency in isolating faulted sections of the distribution system. The OCR protection method takes longer to detect faults, which can delay the clearing of the fault and increase the risk of equipment damage.

Finally, the work also tried to compare the performance of an FFT-THD-based protection method with the MSOGI-THD and SOGI-THD methods. An FFT-THD method is found to be more computationally expensive and requires a significant number of processor cycles. In contrast, the SOGI-THD method is found to significantly reduce fault detection time and provide accurate fault detection while requiring a low computational burden, making it a possible option to be used for improving the reliability and safety of power grids with DGs.

## 7. Conclusions

A comparative study of two THD protection techniques for DS has been proposed in this paper. These techniques are based on the THD of the grid voltages, the estimated amplitude voltages, and the zero-sequence component to define an algorithm to detect, identify, and isolate faults in the grid. The first method employs an MSOGI-THD to obtain the estimated variables, whereas the second method employs a SOGI-THD for the same purpose.

The simulation results in Section 5 showed that, when compared to MSOGI-THD and traditional OCR and DR protection methods, the SOGI-THD technique is the fastest in detecting and isolating faults. The method proved to be effective under various circumstances, including different fault types, DG penetration levels, fault resistances, and fault locations within the studied network.

The study findings demonstrate that the SOGI-THD protection method achieves a faster fault detection response in the protection system. The time response of the SOGI-THD method has been measured to be between 6 and 8.5 ms across all cases examined. In contrast, the MSOGI-THD method showed a fault detection time response that was between 7 and 10 ms. The DR approach exhibited a longer fault detection time, exceeding 15 ms. The OCR approach was found to be undesirable, given its fault detection time of more than 150 ms.

In terms of fault detection accuracy, the SOGI-THD approach outperforms OCRs and DRs. The SOGI-THD has a high detection threshold and the ability to operate regardless of the harmonic distortion of the grid at the moment of the fault. Conversely, OCRs have limited accuracy and may not accurately detect faults under certain conditions, such as high impedance faults. While DRs are more accurate than OCRs, they may require more complex algorithms and higher computational resources.

Compared to other methods, the SOGI-THD approach requires the least computational burden from digital processors. It employs only three SOGIs and 447c for execution, involving few mathematical operations to obtain the estimated variables. In contrast, the MSOGI-THD requires 12 SOGIs and 1788c for execution, while the FFT-THD needs 21057c. This indicates that SOGI-THD is the most computationally efficient method.

Furthermore, the practical advantages of the SOGI-THD method were demonstrated in terms of the trip time response speed of the PD when compared to other protection methods that operate under similar conditions.

In future work, the method’s findings can be verified experimentally to ensure their reliability. Moreover, the feasibility of implementing the methods in more complex DSs, such as ring DS, that feature varying high voltage levels can be investigated. Furthermore, further research could be directed toward minimizing the communication requirements within the system.

## Figures and Tables

**Table 1:** Parameters of the system.

Main Grid	HV/MV Transformer (YNd11)	Distribution Lines (DLs)	MV/LV Transformer (Dyn11)	DGs Rating
Rated voltage: 66 kV Short circuit power: 360 MVA	Rated power: 20 MVA Rated Voltage: 66/20 kV U_sc_ (%): 11	Resistance: 0.16 Ω/km Reactance: 0.109 H/km Capacitance: 0.31 μF/km Length of line: 2 km	Rated power: 400 kVA Rated Voltage: 20/0.4 kV U_sc_ (%): 4.5	Rated power: 6 MVA Rated Voltage: 400 V

**Table 2:** Some of the cases utilized in testing the protection methods.

Fault Type	Location	Defined as	Contribution of DG	Extra Communication Delay	Harmonics Previous to the Fault	
3PH-G	DL1	F1	DG1 = 6 MW DG2 = 6 MW	25 ms	No	Zero
2PH	DL3	F2	DG1 = Zero DG2 = Zero	Zero	Yes	5% of 5th Harmonic

**Table 3:** OCR Settings.

Parameter	Value
Pick up current (pu)	1
Time dial (TD)	0.5
Current transformer ratio (CT)	500:1

**Table 4:** DR Settings.

Parameter	Value
Differential current (pu)	1.08
Biased characteristic (K)	0.5
Current transformer ratio (CT)	500:1

**Table 5:** Fault detection and clearing times of the protection approaches with a 3PH-G fault at F1.

Approach	Fault Detection Time (ms)	Fault Clearing Time (s)
SOGI-THD	6.0	0.3060
MSOGI-THD	9.0	0.3090
DR	15.0	0.3150
OCR	175.0	0.4750

**Table 6:** Number of SOGIs and cycles required to compute the THD of the three-phase system in each method.

Method	Number of SOGIs	Number of Cycles (c)
SOGI-THD	3	447
MSOGI-THD	12	1788
FFT-THD	Not applicable	21,057

**Table 7:** Comparison of different protection approaches operating under similar conditions.

References	Protection Strategies	Parameter Used	Speed	Accuracy	Cost	Inverter Based	Communication	Grid Configuration
[25]	Wavelet individual entropy and fuzzy inference system	Current	High	High	Low	Yes	Not Required	Radial
[28]	fuzzy logic technique	Current	High	Medium	Low	No	Not Required	Radial/Ring
[26]	Wavelet transform and support vector mechanism	Voltage	Medium	High	High	No	Not Required	Radial/Ring
[29]	Statistical morphology, recursive least square methods and Butterworth filter	Current	High	High	High	Yes	Required	Ring
[31]	Differential phase angle criteria	Voltage	High	High	High	Yes	Required	Ring
[32]	S-transform	Current and Voltage	Medium	Medium	High	Yes	Required	Radial
[33]	Power spectral density and transform.	Current	High	High	High	Yes	Not Required	Radial
[34]	Deep belief network, time-time transform and PUM	Current	Low	High	High	Yes	Required	Radial/Ring
[27]	Traveling wave and wavelet analysis	Current	High	High	High	No	Required	Radial
[30]	Least square Adaline algorithm and modified support vector mechanism	Current	Medium	High	High	Yes	Not Required	Radial/Ring
[35]	Hilbert-Huang Transform differential relay	Current	High	High	High	Yes	Required	Ring
[37]	SOGI-THD	Voltage	High	High	Low	Yes	Required	Radial

**Table 8:** Comparison of the THD approaches with other methods operating under similar conditions.

References	Protection Strategies	Trip Time	Advantages	Disadvantages
[59]	Centralize Controller and Linear Programming	421 ms	Relay settings can be obtained instantly without requiring any training	Possibility of communication failures, the complexity grows with a higher number of buses
[60]	Multi-Agent System and OCR	300 ms	No central controller	Possibility of communication failures
[61]	OCR	>200 ms	Variable fault resistance and DG penetration	Not adaptable for network modifications
[62]	Dual settings OCR	>100 ms	Variable fault resistance and DG penetration	Offline calculation
[63]	Multi-Terminal DR	90 ms	Fast, variable fault resistance and DG penetration	Possibility of communication failures
[23]	THD	20–50 ms	Fast, no need for a voltage transformer	Possibility of communication failures, validated only for three-phase faults
[64]	Deep neural network	20–30	Fast, variable fault resistance and DG penetration	Possibility of communication failures and inflexibility in the case of network modifications
[16]	OC and ANN	14 ms	Fast, variable fault resistance and DG penetration	Possibility of communication failures, a complex training process, and inflexibility in the case of network modifications
[36]	MSOGI-THD	7–10 ms	Fast tripping, variable fault location and types, variable fault resistance and DG penetration, affordable computational burden.	Possibility of communication failures
[37]	SOGI-THD	6–8.5 ms	The same merits of the MSOGI-THD. In addition, it is faster and more reliable with a higher THD threshold, requires fewer SOGIs, and has a lower computational burden.	Possibility of communication failures.

## Data Availability

No new data were created in this study.
